# The Burden of Comorbidity Between Bipolar Spectrum and Obsessive-Compulsive Disorder in an Italian Community Survey

**DOI:** 10.3389/fpsyt.2020.00188

**Published:** 2020-03-31

**Authors:** Mauro Giovanni Carta, Naomi Fineberg, Maria Francesca Moro, Antonio Preti, Ferdinando Romano, Matteo Balestrieri, Filippo Caraci, Liliana Dell'Osso, Guido Disciascio, Filippo Drago, Maria Carolina Hardoy, Rita Roncone, Luigi Minerba, Carlo Faravelli, Jules Angst

**Affiliations:** ^1^Department of Medical Sciences and Public Health, University of Cagliari, Cagliari, Italy; ^2^Hertfordshire Partnership NHS Foundation Trust, Queen Elizabeth II Hospital, Welwyn Garden City, United Kingdom; ^3^Department of Clinical and Pharmaceutical Sciences, University of Hertfordshire, Hatfield, United Kingdom; ^4^Mailman School of Public Health, Columbia University, New York, NY, United States; ^5^Dipartimento di Sanità Pubblica e Malattie Infettive, University of Roma La Sapienza, Rome, Italy; ^6^Clinica Psichiatrica, University of Udine, Udine, Italy; ^7^Department of Drug Sciences, University of Catania, Catania, Italy; ^8^Oasi Research Institute-IRCCS, Troina, Italy; ^9^Department of Clinical and Experimental Medicine, University of Pisa, Pisa, Italy; ^10^Scuola di Specializzazione in Psichiatria, University of Bari, Bari, Italy; ^11^Psichiatric Unit, Azienda Ospedaliera Brotzu, Cagliari, Italy; ^12^Dipartimento di Medicina Clinica, Sanità Pubblica, Scienze della Vita e dell'Ambiente, University of L'Aquila, L'Aquila, Italy; ^13^Dipartimento di Scienze Formazione e Psicologia, University of Florence, Florence, Italy; ^14^Department of Psychiatry, Psychotherapy and Psychosomatics, Psychiatric Hospital, University of Zurich, Zurich, Switzerland

**Keywords:** obsessive compulsive disorders, bipolar disorder, epidemiology, community survey, bipolar spectrum, quality of life

## Abstract

**Background:** The impact of the comorbidity between Obsessive-Compulsive Disorder (OCD) and Bipolar Disorder Spectrum (BDS) remains to be clarified. The objective of this study was to examine the lifetime prevalence of OCD, the strength of the association of OCD with comorbid BDS and the role of comorbidity of OCD with BDS in the impairment of health-related quality of life (HRQoL) in an Italian community survey.

**Methods:** The study is a community survey. The sample (*N* = 2,267; women: 55.3%) was randomly selected after stratification by sex and four age groups from the municipal records of the adult population of one urban, one suburban, and at least one rural area in six Italian regions. Physicians using a semi-structured interview (Advanced Tools and Neuropsychiatric Assessment Schedule, ANTAS-SCID) made Diagnostic and Statistical Manual of Mental Disorders – 4th revision (DSM-IV) diagnoses of OCD, Major Depressive Disorder (MDD) and Bipolar Disorder (BD). HR-QoL was measured by the Health Survey Short Form (SF-12). Lifetime Hypomania and subthreshold hypomania were screened by the Mood Disorder Questionnaire (MDQ). BDS was defined as the sum of people shown to be positive for hypomania by the MDQ—with or without a mood disorder diagnosis—plus people with a BD-DSMIV diagnosis even if negative for hypomania at the MDQ.

**Results:** Overall, 44 subjects were diagnosed with OCD, 6 with MDD and 1 with BD. The lifetime prevalence of OCD was 1.8% in men (*n* = 18) and 2.0% in women (*n* = 26). MDD with lifetime subthreshold hypomania (i.e., people screened positive at the MDQ, even without diagnosed mania or hypomania at the interview) was associated with OCD (OR = 18.15, CI 95% 2.45–103.67); MDD without subthreshold hypomania (and screened negative at the MDQ) was not (OR = 2.33, CI 95% 0.69–7.01). People with BDS were strongly associated with OCD (OR = 10.5, CI 95% 4.90–12.16,). People with OCD and BDS showed significantly poorer HR-QoL than people with OCD without BDS (*F* = 9.492; *P* < 0.003).

**Discussion:** The study found a strong association between BDS and OCD. BDS comorbid with OCD was associated with more severe impairment of HR-QoL than OCD without comorbid BDS. Identification of symptoms of hypomania, including subthreshold symptoms, may therefore be important in people with OCD as they might predict a course with poorer HR-QoL.

## Introduction

Obsessive-Compulsive Disorder (OCD) is a common, disabling neuropsychiatric disorder known to be highly comorbid with mood, anxiety, and other disorders classified in the “Obsessive-Compulsive And Related Disorders” group (1, 2). Comorbidity with other psychiatric disorders increases the levels of distress and disability, while the association with Bipolar Disorder (BD) is thought to be particularly harmful and difficult to treat (2).

Although there is a considerable body of recent research on OCD, most studies have focused on course, treatment, pathogenesis, and brain imaging, while methodological differences between published epidemiological studies leave many aspects of prevalence, and especially the comorbidity of OCD with mood disorders, in need of clarification (3, 4).

The estimated prevalence of OCD varies across surveys (2), perhaps as a consequence of different approaches in their design and methodology and of the diagnostic method adopted (1, 3, 5). However, the vast majority of studies, including the more recent ones adopting more structured interviews and lay interviewers (3–7) have found lifetime prevalence rates in a range of 2–3%. However, all of these results are far below the OCD findings from the recent WHO Health Survey, which reported a lifetime prevalence of DSM-IV OCD of 6.2%, ranging from 5.8% in low- and middle-income countries to 6.9% in high-income countries (8).

Lifetime OCD occurring in people with lifetime diagnosis of BD range from 11 to 21% in population-based surveys (9, 10). Lower values were found in hospital-based studies, ranging from 3 to 16% (11, 12). It is not known whether this could be due to the lower diagnostic threshold (of one or both of the disorders) in the community surveys combined with higher co-morbidity in sub-threshold cases, or if it is due to higher accuracy of the diagnoses with co-morbidity produced by the use of standardized tools in community surveys. Conversely, there is a wide variance in the reported lifetime prevalence of Bipolar Disorder Spectrum (BDS) in people with OCD, ranging from 6 to 55% depending on the method of investigation and the definition of the bipolar spectrum adopted (12). A recent meta-analysis estimated the lifetime prevalence of OCD in patients with BD to be 10.9% (95% CI: 7.8–14.4%) (13). This estimated lifetime prevalence was several times higher than the prevalence of OCD estimated in the general population (2.5%; 1.9–3.1%) (13).

There are authors who include in their definitions of the bipolar spectrum those patients in which episodes of subthreshold mania occur concomitantly with lifetime depressive episodes, anxiety disorders, obsessive compulsive disorders, and substance abuse disorder but without previous episodes of mania or hypomania (14). However, according to some authors the spectrum also includes the hyperthymic temperament, even without episodes of impaired mood (15). Yet some other authors define the bipolar spectrum as including those symptoms that have been seen to be associated with or to precede the onset of bipolar disorder: sleep disturbances, subthreshold anxiety and depression, behavioral dyscontrol, and irritability (16).

People with both OCD and BD have been found to show greater disability, poorer HR-QoL, and poorer treatment response compared to those with only one of the two disorders (12). However, a similarly negative outcome was also found in patients having OCD in association with conditions of the BDS, i.e., with subthreshold hypomania but without a DSM diagnosis of BD (12).

It should further be noted that in epidemiological studies the approach based on lay interviewers and structured interviews has been of little help in explaining the role of BD, since this method of information gathering hinders the identification of the disorder, particularly BD II (alternating episodes of Major Depressive Disorder [MDD] and of hypomania). The frequent lack of subjective insight regarding episodes of hypomania means that a correct diagnosis requires sources other than self-reporting (i.e., how these patients appear to others, and what relatives and friends say about the patient) (14). Such information is systematically gathered in clinical practice but is not collected by structured interviews conducted by lay interviewers in epidemiological surveys. This approach has led to the frequent mis-identification of episodes of the depressive phase of the BD as a part of a MDD (14).

This issue is particularly relevant for the study of the comorbidity of OCD with mood disorders. Data from the Zurich study (17) have underlined a closer association between OCD and BD (but also with the subthreshold bipolar syndromes) than between OCD and MDD. The Zurich study was the only epidemiological survey repeated over time (which is an additional source for identifying lifetime mania) and also the only one, in recent years, in which clinicians made the diagnoses. That study further indicated that comorbidity with BD (and with subthreshold bipolar syndromes) is a powerful factor in determining impairment in people suffering from OCD (2).

The objectives of this paper are therefore to extend the investigation of the association between OCD and bipolar spectrum by (a) identifying the lifetime prevalence of OCD in a community study in six Italian regions, using semi-structured interviews conducted by physicians with experience in mental health; (b) determining the comorbidity of OCD with mood disorders, both MDD and BD, including subthreshold manic episodes (BDS broadly defined); and (c) clarifying how far OCD in association with MDD, BD, and subthreshold manic episodes impairs HR-QoL.

## Methods

### Design: Community Survey

#### Sample and Setting

The sample was randomly drawn from the municipal records of one urban, one suburban, and at least one rural area in six Italian regions, which were chosen because they are representative, in terms of geography and socio-cultural characteristics, of the entire national territory. Randomization for those registers was performed after stratification by sex and four age groups (18–24; 25–44; 45–64; >64). Participants were contacted by phone and mail. The interviews were carried out by physicians, face to face in the subjects' homes. The survey methodology was published earlier (18). The main objective of that study was to measure the relationship between the use of drugs and psychiatric disorders in the community.

### Tools

1) The Advanced Tools and Neuropsychiatric Assessment Schedule -Structured Clinical Interview for DSM-IV (ANTAS—SCID-IV) (18), a semi-structured, clinician administered, computerized interview derived from the SCID (19); ANTAS–SCID-IV allows diagnosis of Mood, Anxiety and Eating disorders according to DSM—IV-TR (20) high reliable with SCID (18).
2) The Italian version (21) of the Mood Disorder Questionnaire (MDQ) (22) was adopted to assess lifetime hypomanic symptoms and subthreshold hypomanic episodes. Although this tool shows low accuracy as a screener for DSM—defined BD (23), the research has shown that it is acceptable for identifying subthreshold cases (15).
3) The cut-off score for screening positive for BDS was established on the basis of the standardization study (21) and involved scoring “Yes” to ≥8 items on a list of the 13 best known symptoms of mania / hypomania and “yes” to a question “have several of these ever happened during the same period of time?”
4) The Health Survey Short Form (SF−12) (24) was used to assess perceived HR-QoL as a measure of the impact of OCD and its comorbid conditions. The SF-12 covers physical functioning, physical health, emotional state, physical pain, general health, vitality, social activity, and mental health. The HR-QoL is considered a relevant construct for measuring outcome in chronic diseases that require long-term treatments and greatly impair and impact daily life (25).

### Definitions and Subgroups

OCD, MDD, and BD were defined according to DSM-IV criteria.

The BDS group was defined as including either all those with DSM-IV BD (i.e., diagnosed with Bipolar Disorder on the ANTAS-SCID) and all those screening positive on the MDQ. This group thus includes those diagnosed (on the ANTAS-SCID) with BD or with MDD and lifetime hypomanic symptoms (i.e., MDQ positives), and those with lifetime subthreshold hypomania (i.e., MDQ positives) but having no diagnosis of MDD or BD according ANTAS-SCID.

The MDD group was thus sub-divided into those with and without subthreshold lifetime hypomania (i.e., respectively, screening positive or negative on the MDQ) and included in or excluded from the BDS group.

### Statistical Analysis

The odds ratio (univariate analysis) for DSM-IV OCD diagnosis and age, gender, and comorbidity with DSM-IV MDD, DSM-IV BD, and BDS (i.e., any case diagnosed with BD according to DSM-IV or screened positive on the MDQ) was calculated using one of the groups in each table as a “pivot.” Statistical significance was calculated using the χ^2^ test. Odds, ratios and 95% confidence intervals were calculated using Miettinen's simplified method (26). The comparisons between the scores on the SF-12 in the different groups were calculated by one-way ANOVA.

The burden in the worsening of HR-QoL attributable to OCD was calculated as the difference between the HR-QoL (mean score on the SF-12) in a sample from the same community survey database of people *without* OCD and the SF-12 mean score of those *with* OCD. The randomization was conducted after standardization by blocks. For each person with OCD two people, matched by age and sex, were selected by randomization from the cell of all without OCD.

A similar method was adopted for measuring the burden in the deterioration of HR-QoL attributable to OCD with BDS. Thus, the attributable burden of OCD *with* BDS was the difference between the HR-QoL (mean score on the SF-12) in a sample from the community survey database of people *without* OCD and the SF-12 mean score of those *with* OCD plus BDS as above defined (BD + all positives on MDQ). The proportion of the sample drawn for comparison (without OCD) was 2/1 for OCD without BDS and 4/1 for OCD with BDS.

## Results

Overall, 44 subjects were diagnosed with OCD at the ANTAS-SCID, 6 were diagnosed with MDD and just 1 was diagnosed with BD.

Table 1 illustrates the lifetime prevalence for OCD by age and gender in the epidemiological sample (2,267 subjects, 1,253 women [55.3%]). The prevalence of OCD was 1.8% in men (*n* = 18) and 2.0% in women (*n* = 26); with no difference by gender (OR = 1.15, CI 95% 0.61–2.19). The prevalence rate among young women aged 18–24 (*n* = 7 [5.1%]), was higher than in all other age groups.

**Table 1:** Lifetime Prevalence of OCD by sex and age.

	***N* (Subtotal)**	**Prevalence (%)**	**χ^2^ 1df**	***P***	**OR**	**CI 95%**
<25 Men	2 (133)	1.5	–			
25–44	8 (381)	2.1	0.01^°^	0.952	0.71	0.10–3.68
45–64	5 (312)	1.6	0.01^°^	0.999	0.93	0.12–5.49
>64	3 (188)^°^	1.6	0.01^°^	0.999	1.01	0.25–4.31
Total men^*^ [43.4%]	18 (1014)	1.8				
<25 Women	7 (137)	5.1	—			
25–44	7 (437)	1.6	5.71	0.017	3.41	1.01–11.01
45–64	9 (429)	2.1	4.99	0.046	2.63	1.00–7.13
>64	3 (250)	1.2	5.60	0.018	4.55	1.01–22.6
Total women^*^ [56.6%]	26 (1253)	2.0	0.209	0.647	1.15	0.61–2.19
Total (men + women)	44 (2267)	1.9				

* *Female vs. Males*.

° *with Yates's correction*.

Table 2 shows the associations between mood disorders and OCD. MDD was associated with OCD in 13.6% of all cases of OCD (OR = 3.69, CI 95% 1.36–9.41). When the set of MDD was subdivided into those cases with and without subthreshold episodes of hypomania (MDQ positive and MDQ negative), only the subgroup of MDD with lifetime subthreshold hypomania, constituting 4.5% of the whole OCD sample, showed a strong association with OCD (OR = 18.15, CI 95% 2.45–103.67). In contrast, the MDD subgroup without subthreshold hypomania, constituting 9% of the whole OCD sample, showed no association with OCD (OR = 2.33, CI 95% 0.69–7.01). The BDS group (as defined above) was also strongly associated with OCD (OR = 10.5, CI 95% 4.90–12.16). The one participant who was recognized positive for BD at the ANTAS-SCID also had OCD (2.8%; OR = 26.6, CI 95% 0.93–384.3).

**Table 2:** Comorbidity between Obsessive Compulsive Disorders and Major Depressive Disorders (MDD) with further subdivision between MDD screening positive on the MDQ and MDD screening negative on the MDQ; Bipolar Disorders and Bipolar Spectrum Disorders (including BD, MDD positives on the MDQ, and positives on the MDQ without a diagnosis of BP or MDD).

	***N***	**Comorbidity with OCD (%)**	**χ2**	***p***	**OR**	**CI 95%**
Major Depressive Disorder	6 (44)	13.6	9.58	0.002	3.69	1.36–9.41
Major Depressive Disorder positives on MDQ	2 (44)	4.5	12.37^*^	<0.0001	18.15	2.45–103.67
Major Depressive Disorder negatives on MDQ	4 (44)	9.0	2.67	0.102	2.33	0.69–7.01
Bipolar Disorder	1 (44)	2.8	3.55^*^	0.059	26.6	0.93–384.3
All positives on MDQ = Bipolar Spectrum (Bipolar Disorder + MDD/MDQ+ + MDQ+ without diagnosis of MDD or BP)	12 (44)	27.2	65.49^*^	<0.0001	10.5	4.90–12.16

* *With Yates's Correction*.

*MDQ+ = screening positive on the MDQ*.

In terms of the impairment of HR-QoL attributable to OCD alone, OCD with BDS and OCD without BDS, the results revealed that OCD alone is a significant factor in compromising HR-QoL, with mean SF-12 scores of 35.4 ± 6.9 and an attributable impairment of HR-QoL of 2.9 ± 6.0 in 44 cases as against 88 controls. We isolated the 12 cases of OCD with BDS (of these 12 cases, one also had a diagnosis of BD, 6 had a diagnosis of Panic Disorder, 2 of MDD [1 of which had comorbid Panic Disorder], 1 of Post Traumatic Stress Disorder). These cases were found to have a significantly higher level of impairment of HR-QoL than the other OCD cases, even though the latter group included those with MDD screening negative on the MDQ. The mean SF-12 scores were 33.6 ± 6.7 in those with OCD and BDS, and it 36.1 ± 7.1 in those with OCD without comorbid BDS, with an attributable impairment of HR-QoL of, respectively, 6.1 ± 5.9 (12 cases vs. 48 controls) and 2.1 ± 7.4 (32 cases vs. 64 controls): F_[1;110;111]_ = 9.49; *P* < 0.003.

## Discussion

The study, which was based on semi-structured clinical interviews administered by physicians with experience in mental health, found lower prevalence rates of OCD than the recent WHO epidemiological survey (8). Our results were closer to other studies conducted since the 1980s, including those relying on lay interviewers and structured interviews (3, 5, 27–31), although caution is needed when comparing these surveys on account of methodological differences.

Our study showed a higher frequency among younger age groups, which suggests a youthful onset or a cohort effect (8).

The frequency of the association between Bipolar Disorder and OCD in our sample (2.8%) is lower than the frequency found in previous epidemiological studies (10, 11) but in the range [2–3%] reported in the aforementioned clinical studies (11, 12), and in studies looking for comorbidity with BD in patients with OCD (32); this is probably due to methodological differences. The diagnosis of BD (and OCD) in our study was detected with a semi-structured interview conducted by clinicians, as opposed to the community surveys in which the diagnoses are made by structured interviews conducted by lay interviewers. It is known that the frequencies of psychiatric disorders identified with the SCID interview are tendentially low (14). Studies form the same data-base of the present found lower frequencies of both MDD (18) and BD (33) compared to other studies conducted by means of structured interviews.

The notable finding of the present study is the close association between OCD and BDS including DSM-IV Bipolar Disorder, DSM-IV Major Depressive Disorder showing hypomanic subthreshold episodes, and people showing hypomanic subthreshold episodes without a mood diagnosis. The association of OCD with the BDS is well-documented in clinical settings (12, 34).

Our study also found that in a random sample of the general population the comorbidity between OCD and BDS is associated with a seriously impaired HR-QoL. That impairment is considerably less marked in people with OCD who did not display bipolar spectrum symptoms. The impairment of HR-QoL attributable to the comorbidity of OCD with the BDS is substantial, and the measurable burden is equal or very similar to that of other psychiatric (MDD, Panic Disorder, Eating Disorders) conditions, as measured in case-control studies that used the same methodology applied in the present study (35–38). It should be noted that some of the cases included in the BDS subgroup also were comorbid with other disorders, such as Panic Disorder, and this might have influenced the extent of the impairment of HR-QoL attributable to the comorbidity of OCD. However, BD is highly comorbid with anxiety disorders such as Panic Disorder (39), and therefore it might be difficult to disentangle the impact of the associated hypomania and of comorbid anxiety disorders when investigating samples that are distinguished on the basis of their comorbidity with BD.

This finding is consistent with the clinical observation that cases of OCD in association with BD (including subthreshold manifestations) are the most severe and have a worse course (40, 41). The prospective epidemiological Zurich Study, which analyzed two groups of probands from age 20 to 40, one having OCD with BD, the other without, showed more chronic episodes, residual symptoms and previous recurrence of depressive episodes in the comorbid group. In the comorbid group higher rates of anxiety disorders, impulse control disorders, eating disorders, and tic disorders were also found (17). The follow-up analysis to age 50 also confirmed that the co-occurrence of OCD and bipolar spectrum (including subthreshold bipolar disorder) was associated with significantly higher levels of treatment-seeking, impairment, distress, suicidality, and an increased risk of alcohol abuse/dependence compared with “pure” OCD (2), although this is not an universal finding (42). A poorer quality of life was also indicated as a specific feature of bipolar-OCD comorbidity (41). In agreement with these results, our study found that the association of OCD with BDS produced more substantial impairment than those with comorbid MDD but without bipolar spectrum symptomatology. Given that a diagnosis of MDD is known to be a major determinant of reduced HR-QoL when comorbid with other psychiatric conditions (18), this finding emphasizes the damaging effect of BDS on HRQoL, with or without diagnosis of a mood disorder, when combined with OCD.

Criticism has been leveled against the use of screening questionnaires to detect cases of BD. It is argued that only a small percentage of “positives” on such screening tools would be recognized as having BD if they were to undergo a structured interview using DSM as the diagnostic gold standard (23, 43, 44). However, our research has provided clear evidence that the wide spectrum of “false positives” that emerge when screening for BDS (i.e., being positive at screening but below the diagnostic threshold according to the gold standard international descriptive criteria) show demographic characteristics, a comorbidity profile, a type of social impairment, drug and health care use, and have brain imaging profiles that indicate that they belong to clearly defined subthreshold bipolar spectrum group (14, 18). It is also interesting to note that in the previously mentioned clinical studies on OCD, many of the characteristics identified as defining OCD comorbid with bipolar subthreshold, namely anxiety disorders, substance abuse, personality disorders, and the risk of suicide, are also to be found among the so-called “false positives” identified by screening tools such as MDQ, i.e., people positive at the screening but who did not fulfill the DSM-IV criteria for BD (14).

The results suggest that screening people with OCD for symptoms of hypomania is crucial, as the comorbidity represents an important marker of severity and risk of impairment. The fact that longitudinal clinical studies have also found that the presence of hypomanic symptoms (including subthreshold symptoms) is a predictor of treatment response (45–47) adds strength to the argument.

## Strengths and Limitations

This study has both strengths and limitations. The main strengths are the use of structured, standardized instruments for measurement, and the investigation in a reasonably large, community-based sample randomly drawn from the municipal records of geographical areas expected to be representative of the socio-cultural characteristics of the entire national territory.

The most important limitation is that, although the research is a community survey with a large sample, the focus of this research was not the main objective of the project. The sample was built on the basis of the expected frequencies of subthreshold mania (focus of main objective of the whole project), which was estimated at 4% (18), while the lifetime prevalence of OCD (focus on this secondary objective) was found in this study to be <2%. Thus, the hypotheses that can arise from the reading of these data have a heuristic and stimulating value rather than a conclusive one. The results must therefore be confirmed by future studies with larger samples and specifically focused on this topic.

Another important limitation is that we used ratings on the MDQ to identify a diagnostic group (“subthreshold bipolar disorders”) that is hard to pin down and is particularly controversial. Additional limitations include the lack of details on some correlates of BDS comorbidity among OCD participants, which could be of interest on a clinical ground (e.g., OCD course and severity, symptom dimensions, suicidality, other comorbidities); lack of statistical power for some analysis due to the small size of some subsamples; lack of information on some unexplored comorbidities, such as impulse control disorder or personality disorders, which could have a differential impact on HR-QoL in the two study groups.

## Conclusions

These results from a random sample of the general population have found a strong association between mood disorders including bipolar and subthreshold bipolar symptomatology and the diagnosis of OCD. These findings confirm that cases of BDS comorbid with OCD are associated with more severe impairment of quality of life. The identification of symptoms of hypomania, including subthreshold symptoms, may therefore be crucial in people with OCD. The results must be confirmed by future studies.

## Data Availability Statement

The datasets for this article are not publicly available because: the agreement shared with the partners in the planning of the study, in the presentation for the assignment of the original grant and in the request for authorization to the ethics committee was that the database (with anonymous records) would be available under the review of the project leader as guarantor. Requests to access the datasets should be directed to Mauro Giovanni Carta.

## Ethics Statement

The ethics committee of the Italian National Health Institute, Rome (Istituto Superiore della Sanità) approved the study. All procedures performed in the studies were in accordance with the ethical standards of the institutional and/or national research committee and with the 1964 Helsinki declaration and its later amendments. Each participant was provided with full information on the aims and methodology of the study and signed an informed consent form. Participants were also made aware of data protection and privacy laws and that they could terminate the interview at any time. It was explained that the data would be used as an anonymous database, in order to maintain confidentiality in accordance with data protection laws. In addition, all studies that are carried out by our Center at the University of Cagliari are reviewed by the Comitato Etico Indipendente Azienda Ospedaliera Universitaria di Cagliari. Written informed consent was collected from all participants.

## Author Contributions

MC had the first idea of the epidemiological study that was planned and carried out jointly with MB, FC, LD, GD, FD, MH, RR, and CF (these researchers are also responsible for the database). The present study was proposed by the MC, JA, and MM to the coordination group. The processing group was also involved, due to their specific skills, NF, FR, AP, and LM. The study and data processing was planned and conducted with the participation of the entire group. MC with MM wrote the first draft that was revised by each of the participants who approved the reworked paper based on the suggestions.

### Conflict of Interest

The authors declare that the research was conducted in the absence of any commercial or financial relationships that could be construed as a potential conflict of interest.
